# Supplementary material for: Multimodal in vivo recording using transparent graphene microelectrodes illuminates spatiotemporal seizure dynamics at the microscale
Source: Commun Biol. 2021 Jan 29;4:136. doi: 10.1038/s42003-021-01670-9 (PMC7846732; doi:10.1038/s42003-021-01670-9)
Supplement: Supplementary file 2 — Supplementary Information [file 42003_2021_1670_MOESM2_ESM.pdf]

Multimodal *in vivo* recording using transparent graphene microelectrodes illuminates spatiotemporal seizure dynamics at the microscale

Nicolette Driscoll[1,2,3 †], Richard Rosch[1,4,5 †], Brendan B. Murphy[1,2,3], Arian Ashourvan[1,2], Ramya Vishnubhotla[6], Olivia O. Dickens[7], A.T. Charlie Johnson[6], Kathryn A. Davis[2,8], Brian Litt[1,2,8], Danielle S. Bassett[1,6,8,9,10,11], Hajime Takano[8,12] *, and Flavia Vitale[2,3,8,13] *

1. Department of Bioengineering, University of Pennsylvania, Philadelphia, PA, 19104, USA
2. Center for Neuroengineering and Therapeutics, University of Pennsylvania, Philadelphia, PA, 19104, USA
3. Center for Neurotrauma, Neurodegeneration, and Restoration, Corporal Michael J. Crescenz Veterans Affairs Medical Center, Philadelphia, PA, 19104, USA
4. MRC Centre for Neurodevelopmental Disorders, King's College London, London, WC2R 2LS, UK
5. Department of Paediatric Neurology, Great Ormond Street Hospital for Children NHS Foundation Trust, London, WC1N 3JH, UK
6. Department of Physics and Astronomy, University of Pennsylvania, Philadelphia, PA, 19104, USA
7. Graduate Group in Biochemistry and Molecular Biophysics, University of Pennsylvania, Philadelphia, PA, 19104, USA
8. Department of Neurology, Perelman School of Medicine, University of Pennsylvania, Philadelphia, PA, 19104, USA
9. Department of Psychiatry, Perelman School of Medicine, University of Pennsylvania, Philadelphia, PA, 19104, USA
10. Department of Electrical & Systems Engineering, University of Pennsylvania, Philadelphia, PA, 19104, USA
11. Santa Fe Institute, Santa Fe, NM, 87501, USA
12. Division of Neurology, Children's Hospital of Philadelphia, Philadelphia, PA, 19104, USA
13. Department of Physical Medicine and Rehabilitation, University of Pennsylvania, Philadelphia, PA, 19104, USA

[†] These authors contributed equally to this work.
* corresponding authors

Supplementary Information

Supplementary Methods

Optimization of graphene microelectrode array fabrication

Our graphene device fabrication follows closely the protocol outlined by Park et al. in 2016, with several important modifications[1]. Firstly, monolayers of graphene grown on copper foils via chemical vapor deposition (CVD) were transferred to the wafer using a bubble transfer method similar to that described by Gao et. al. in 2012[2]. While conventional graphene transfer methods rely on complete etching of metal substrates in suitable etchants, which causes damage to the graphene and leaves metal residues, bubble transfer using electrolysis of water enables transferring large-area graphene films with high purity and without inducing cracking or damage. Initially, we fabricated devices with either one or two stacked monolayers of graphene to assess the relationship between number of graphene layers and electrochemical impedance (Supplementary Fig. S2a). While we found that adding a second layer of graphene reduced the impedance (2.16 ± 0.23 MΩ for 2-layer vs. 2.75 ± 0.26 MΩ for 1-layer graphene at

1 kHz), we sought to reduce the impedance further without adding additional graphene layers, as these would reduce the optical transparency of our devices. To this end, we explored chemical doping methods for enhancing the out-of-plane conductivity of graphene.

Exposing graphene to nitric acid (HNO_3) results in the adsorption of electropositive NO_3^- groups onto the graphene surface. Specifically, the HNO_3 molecule physisorbs onto the graphene sheet, without breaking any C-C bonds, and then dissociates into three groups: two radicals NO_2^0 and NO_3^0, and a water molecule: 2HNO3 = NO_2^0 + NO_3^0 + H_2O. The two radicals have a singly occupied state below the Fermi energy of the graphene layer, which allows two electrons to transfer from graphene into these states, creating two holes and causing p-type doping[5]. This p-type or hole doping has been shown to reduce electrochemical impedance and improve the noise characteristics of graphene electrodes[3–5]. We optimized a process for HNO_3 doping of graphene, and performed this doping layer-by-layer after the transfer of each graphene sheet to the wafer substrate. Layer-by-layer doping has been shown to produce doped graphene sheets with reduced and more stable sheet resistance values compared to doping after stacking of graphene monolayers, where only the topmost layer experiences doping[3, 6]. To assess the performance of devices prepared under different conditions, we compare impedance values both at the commonly-used 1 kHz reference frequency, and at the lower frequency of 100 Hz, since many physiologic signals of interest in the brain are of lower frequency content than the typical 1 kHz reference frequency (Supplementary Fig. S2b,c). We find that the impedance relationships between 1-layer, 2-layer, and doped 2-layer graphene devices are similar at 1 kHz and 100 Hz, with the advantage of chemical doping being more pronounced at lower frequencies: the 100 Hz impedance of 1-layer devices is 25.6 ± 11 MΩ, 2-layer devices is 15.9 ± 5.5 MΩ, and HNO_3-doped devices is 2.07 ± 0.65 MΩ. The significant spread of 100 Hz impedance values for the un-doped device preparations can be attributed to their high impedance in this lower frequency range, which limits the accuracy of the EIS measurement. As a low electrode impedance is essential for obtaining recordings with high signal-to-noise ratio (SNR)[7, 8], these results indicate that HNO_3 doping is a useful approach for improving the performance of graphene microelectrodes, especially at lower frequencies where most physiologic signals of interest occur. In addition to EIS measurements, cyclic voltammetry (CV) measurements were taken to assess the effect of HNO_3 doping on the charge storage capacity of graphene electrodes. Doping with nitric acid increased the charge storage capacity from 22.87 μC/cm^2 to 64.44

μC/cm^2, indicating that this method of doping may also improve the performance of graphene electrodes for neural stimulation applications (Supplementary Fig. S2d).

Supplementary Figures:

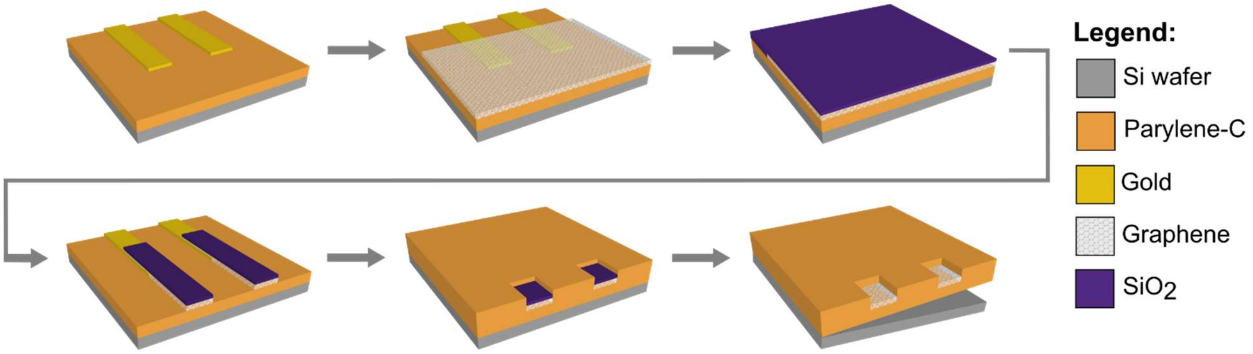

Supplementary Fig. S1 | Fabrication schematic for transparent graphene micro-ECoG. Details of the fabrication are given in the Methods section. Briefly, parylene-C is deposited onto a Si wafer and Ti/Au traces are patterned on the parylene-C. CVD-grown graphene is transferred to this substrate using a bubble transfer method. An SiO_2 layer is deposited to protect the graphene during later processing steps, then the graphene and SiO_2 layers are patterned and etched. A top layer of parylene-C is deposited to encapsulate the device, then VIAs are etched to open the graphene electrode contacts as well as open the Ti/Au bonding pads at the back end of the device. Finally, the SiO_2 covering the graphene electrode contacts is chemically etched away and the devices are removed from the wafer.

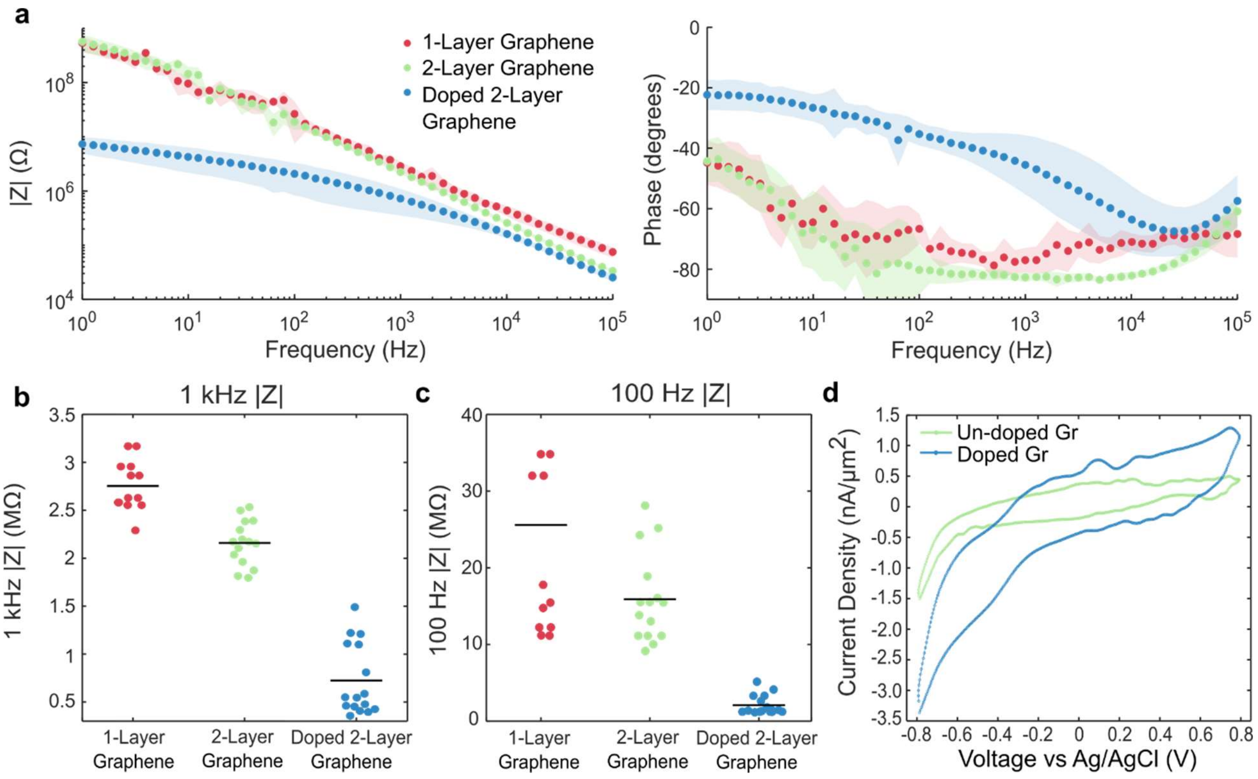

Supplementary Fig. S2 | Optimization of graphene processing. a, Electrochemical impedance spectra for 50 μm x 50 μm graphene μECoG electrodes of three preparations: 1-layer graphene, 2-layer graphene, and HNO_3-doped 2-layer graphene. Impedance magnitude significantly decreased with doping, particularly at frequencies below 1 kHz. Noise in the low-frequency range for the un-doped graphene samples is attributed to the high impedance modulus. Shaded bounds represent standard deviations. **b**, Impedance magnitude at 1 kHz for the three device preparations. Horizontal black bars indicate means. **c**, Impedance magnitude at 100 Hz for the three device preparations. Horizontal bars indicate means. **d**, Cyclic voltammograms showing superior CSC_C in an HNO_3-doped 2-layer graphene electrode, as compared to an un-doped 2-layer graphene electrode (CSC_c is 22.4 μC/cm^2 and 15.9 μC/cm^2, respectively). The scan rate was 500 mV/s.

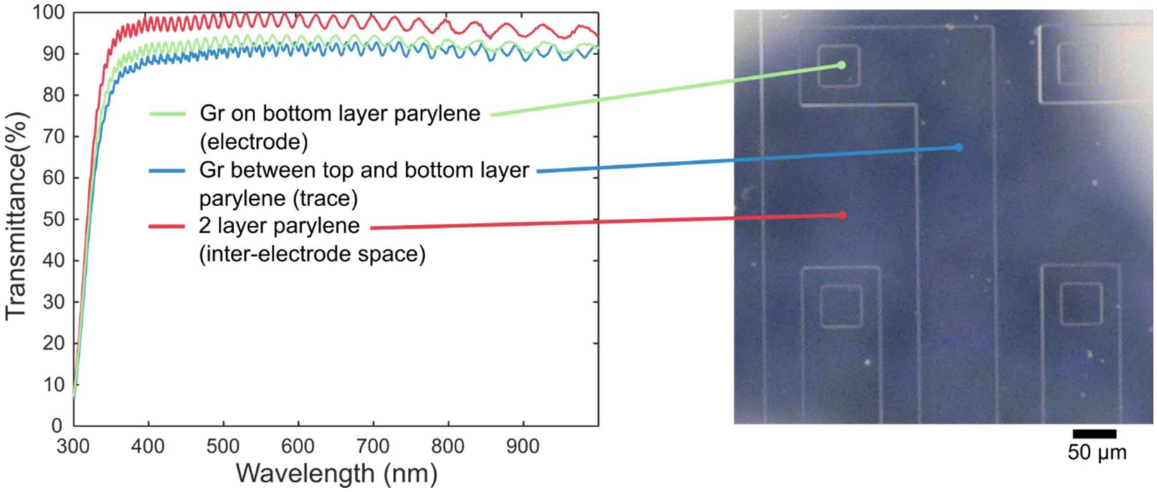

Supplementary Fig. S3 | UV-Visible spectroscopy. Optical transmission spectrum for different locations in the graphene μECoG array. Overall, the device is >90% transparent across the visible to near infrared spectrum. The ringing behavior in the spectra is attributed to the parylene-C.

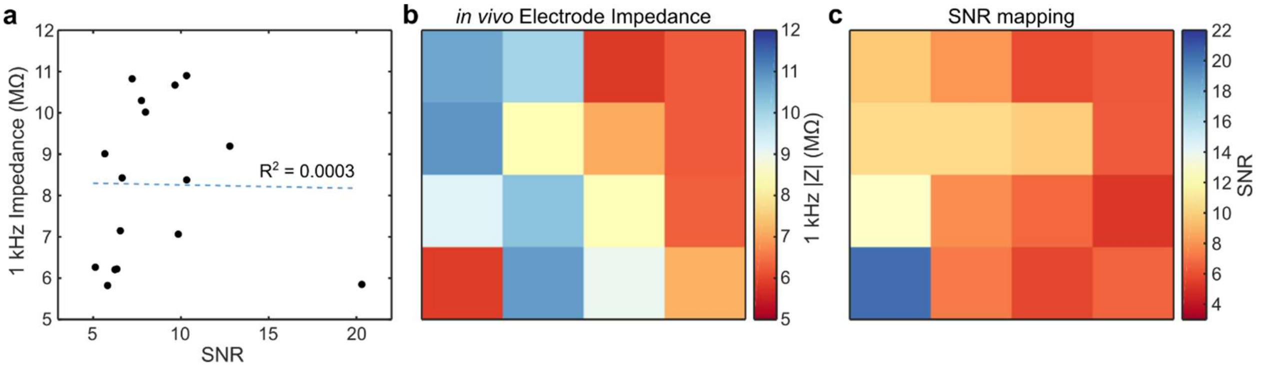

Supplementary Fig. S4 | SNR vs. electrode impedance. **a,** SNR vs. 1 kHz impedance magnitude for each electrode during the *in vivo* recording analyzed in this work. No correlation between SNR and impedance was found, as evidenced by the poor linear fit shown. Note that while the impedance magnitudes measured *in vivo* are significantly higher than those measured in saline, these electrodes still achieve high SNR >5. **b,** Map of *in vivo* electrode impedances across the 16 ch graphene array. **c,** Map of SNR values across the 16 ch graphene array.

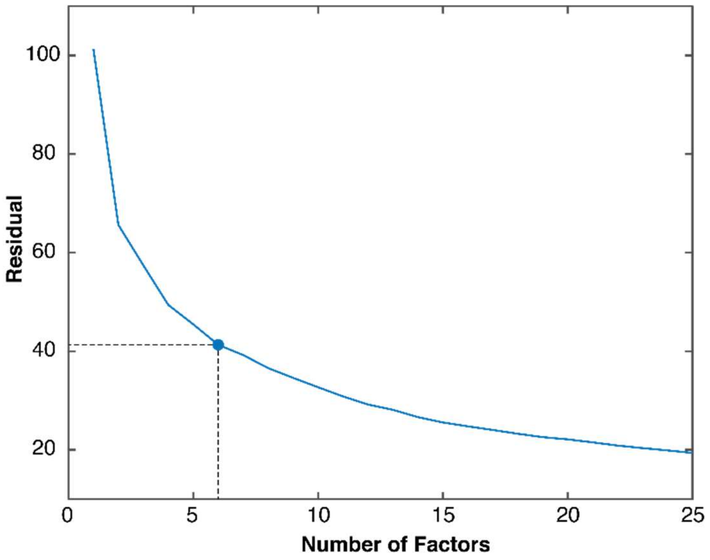

Supplementary Fig. S5 | Variance explained by number of factors. Residuals for NMF fits after optimization for preset factor numbers ranging from 1 to 25. The final number of factors ($\boldsymbol{k}$ = 6) was selected as a trade-off between maintaining a low number of factors and achieving a low residual variance.

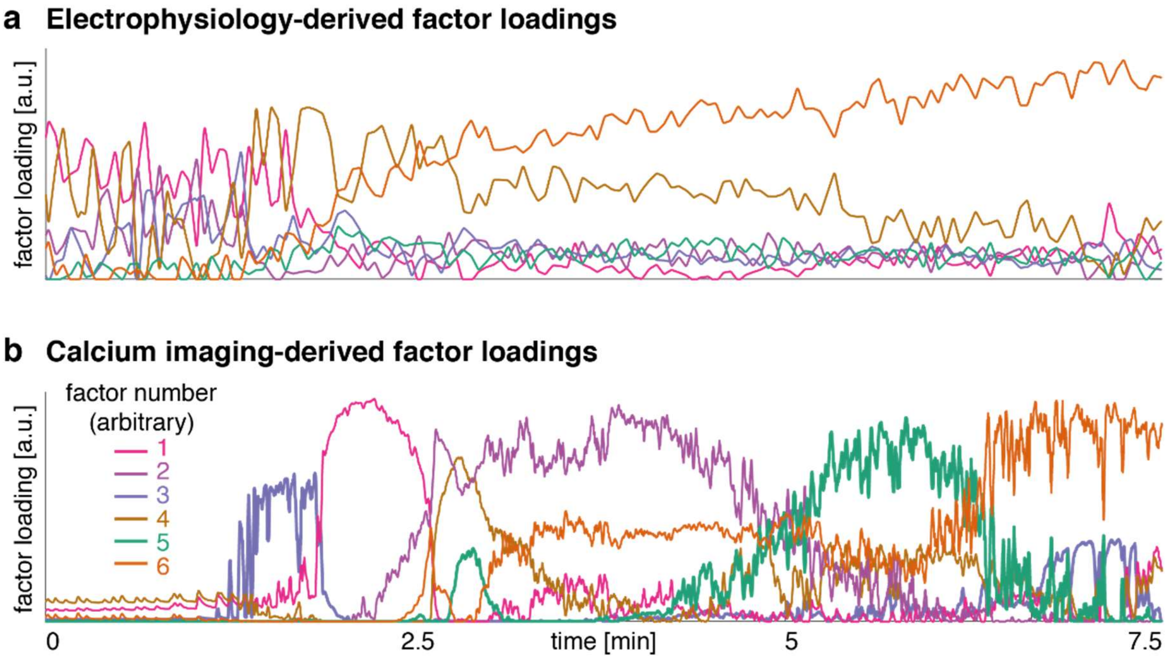

Supplementary Fig. S6 | Factor loadings (i.e. ***H***-matrix) derived from non-negative matrix factorization. **a,** Results derived from matrices containing electrophysiology-derived data features only show transition into seizure and some progressive changes, but no clear sequential states. **b,** Factor loadings derived from calcium imaging features only shows sequential states similar to those of the combined analysis presented in the main text.
