# Supplementary material for: Multimodal in vivo recording using transparent graphene microelectrodes illuminates spatiotemporal seizure dynamics at the microscale
Source: Commun Biol. 2021 Jan 29;4:136. doi: 10.1038/s42003-021-01670-9 (PMC7846732; doi:10.1038/s42003-021-01670-9)
Supplement: Supplementary file 3 — Description of Additional Supplementary Files [file 42003_2021_1670_MOESM3_ESM.pdf]

Description of Additional Supplementary Files

File name: Supplementary Video S1 | Multimodal recording during seizure onset.

Description: Onset and spread of the seizure activity analyzed in Fig. 3 – Fig. 6. Here, we show the normalized fluorescence video (right), and the relevant data for one selected electrode (blue): fluorescence intensity beneath the electrode, LFP bandpower, spectrogram, and raw signal.
