# Supplementary material for: Multimodal in vivo recording using transparent graphene microelectrodes illuminates spatiotemporal seizure dynamics at the microscale
Source: Commun Biol. 2021 Jan 29;4:136. doi: 10.1038/s42003-021-01670-9 (PMC7846732; doi:10.1038/s42003-021-01670-9)
Supplement: Supplementary file 5 — Reporting Summary [file 42003_2021_1670_MOESM5_ESM.pdf]

nature research

Corresponding author(s): Flavia Vitale

Last updated by author(s): Nov 30, 2020

Reporting Summary

Nature Research wishes to improve the reproducibility of the work that we publish. This form provides structure for consistency and transparency in reporting. For further information on Nature Research policies, see our Editorial Policies and the Editorial Policy Checklist.

Statistics

For all statistical analyses, confirm that the following items are present in the figure legend, table legend, main text, or Methods section.

n/a	Confirmed	
☐	☒	The exact sample size (n) for each experimental group/condition, given as a discrete number and unit of measurement
☐	☒	A statement on whether measurements were taken from distinct samples or whether the same sample was measured repeatedly
☐	☒	The statistical test(s) used AND whether they are one- or two-sided<br>*Only common tests should be described solely by name; describe more complex techniques in the Methods section.*
☒	☐	A description of all covariates tested
☐	☒	A description of any assumptions or corrections, such as tests of normality and adjustment for multiple comparisons
☐	☒	A full description of the statistical parameters including central tendency (e.g. means) or other basic estimates (e.g. regression coefficient) AND variation (e.g. standard deviation) or associated estimates of uncertainty (e.g. confidence intervals)
☒	☐	For null hypothesis testing, the test statistic (e.g. F, t, r) with confidence intervals, effect sizes, degrees of freedom and P value noted<br>*Give P values as exact values whenever suitable.*
☒	☐	For Bayesian analysis, information on the choice of priors and Markov chain Monte Carlo settings
☒	☐	For hierarchical and complex designs, identification of the appropriate level for tests and full reporting of outcomes
☒	☐	Estimates of effect sizes (e.g. Cohen's d, Pearson's r), indicating how they were calculated

Our web collection on statistics for biologists contains articles on many of the points above.

Software and code

Policy information about availability of computer code

Data collection	Electrophysiology was obtained using NeuroNexus SmartBox software. Imaging was obtained using HCImage software from Hamamatsu Photonics.
Data analysis	Data analysis was performed using custom scripts in MATLAB R2019a. All scripts to reproduce analysis figures are made available online on: https://doi.org/10.5281/zenodo.4050520

For manuscripts utilizing custom algorithms or software that are central to the research but not yet described in published literature, software must be made available to editors and reviewers. We strongly encourage code deposition in a community repository (e.g. GitHub). See the Nature Research guidelines for submitting code & software for further information.

Data

Policy information about availability of data

All manuscripts must include a data availability statement. This statement should provide the following information, where applicable:

- Accession codes, unique identifiers, or web links for publicly available datasets
- A list of figures that have associated raw data
- A description of any restrictions on data availability

The electrophysiology recorded on the graphene electrode µECoG as well as the calcium fluorescence imaging data are publicly available on https://doi.org/10.6084/m9.figshare.13007840. Figures containing raw data from these datasets include: Fig. 2, Fig. 3, and Fig. 5.

Field-specific reporting

Please select the one below that is the best fit for your research. If you are not sure, read the appropriate sections before making your selection.

☒ Life sciences ☐ Behavioural & social sciences ☐ Ecological, evolutionary & environmental sciences

For a reference copy of the document with all sections, see nature.com/documents/nr-reporting-summary-flat.pdf

Life sciences study design

All studies must disclose on these points even when the disclosure is negative.

Sample size	No sample size calculation was performed for this study. This was an exploratory study to validate new experimental and analytical techniques, performed in a single animal. This sample size is sufficient because we are simply reporting on new methodology, similar to a case study, and we do not make claims about the generalization of these results to additional animals.
Data exclusions	In our analysis, we chose a time period around the onset of the first seizure to analyze in-depth because we were interested in analyzing the transition into the ictal state. We excluded from the analysis the first ~20 seconds of data because the drug 4AP was bath-applied to the exposed cortex during this period, causing large artifacts in the ECoG recording. We did not analyze data beyond the first 8 minutes of recording, because the brain had transitioned into a state of status epilepticus at this point, and we were interested in studying the initial transition of the brain into the seizure state.
Replication	We have reproduced these findings in subsequent animals.
Randomization	There were no separate experimental groups in our study, thus randomization was not relevant.
Blinding	There were no separate experimental groups in our study, thus blinding was not relevant.

Reporting for specific materials, systems and methods

We require information from authors about some types of materials, experimental systems and methods used in many studies. Here, indicate whether each material, system or method listed is relevant to your study. If you are not sure if a list item applies to your research, read the appropriate section before selecting a response.

Materials & experimental systems

n/a	Involved in the study	
☒	☐	Antibodies
☒	☐	Eukaryotic cell lines
☒	☐	Palaeontology and archaeology
☐	☒	Animals and other organisms
☒	☐	Human research participants
☒	☐	Clinical data
☒	☐	Dual use research of concern

Methods

n/a	Involved in the study	
☒	☐	ChIP-seq
☒	☐	Flow cytometry
☒	☐	MRI-based neuroimaging

Animals and other organisms

Policy information about studies involving animals; ARRIVE guidelines recommended for reporting animal research

Laboratory animals	6-8 week-old male mice, Th1-GCaMP6s, Jax #025776
Wild animals	The study did not involve wild animals.
Field-collected samples	The study did not involve samples collected from the field.
Ethics oversight	All experiments were performed under a protocol approved by the Children's Hospital of Philadelphia Institutional Animal Care and Use Committee.

Note that full information on the approval of the study protocol must also be provided in the manuscript.

nature research | reporting summary
